# Supplementary material for: Difference in TMPRSS2 usage by Delta and Omicron variants of SARS-CoV-2: Implication for a sudden increase among children
Source: PLoS One. 2024 Jun 13;19(6):e0299445. doi: 10.1371/journal.pone.0299445 (PMC11175390; doi:10.1371/journal.pone.0299445)
Supplement: Attachment — Submitted filename: 0b. Response to Reviewers.docx [file pone.0299445.s007.docx]

**RESPONSE TO REVIEWERS**

[PONE-D-24-05165] Difference in TMPRSS2 usage by Delta and Omicron variants of SARS-CoV-2: Implication for a sudden increase among children.

**LABORATORY PROTOCOL TO ‘PROTOCOLS.IO’**

If applicable, we recommend that you deposit your laboratory protocols in protocols.io to enhance the reproducibility of your results. Protocols.io assigns your protocol its own identifier [DOI] so that it can be cited independently in the future.

Thank you very much for inviting us to ‘Protocols.io’. Absolutely we can recognize the importance to share our laboratory protocols with readers of ‘Protocols.io’. However, we do not have enough time to summarize and checkup our protocols precisely and should submit the revised manuscript as soon as possible. Therefore, we decided not to submit our file of protocols to ‘Protocols.io’ at this time. If the submission of our protocols to ‘Protocols.io’ is indispensable for the revision, please advise us about it.

**JOURNAL REQUIREMENTS:**

When submitting your revision, we need you to address these additional requirements.

1. **PLOS ONE's style.**

Please ensure that your manuscript meets PLOS ONE's style requirements, including those for file naming. The PLOS ONE style templates can be found at:

https://journals.plos.org/plosone/s/file?id=wjVg/PLOSOne_formatting_sample_main_body.pdf and https://journals.plos.org/plosone/s/file?id=ba62/PLOSOne_formatting_sample_title_authors_affiliations.pdf.

For adjusting to PLOS ONE’s style, the revision was made as follows.

- Level 1 and 2 headings are now appeared in Bold type with 18pt or 16pt fonts.
- A Figure is cited as ‘Fig’ instead of ‘Figure’.
- Figure files are saved such as Fig 1.tiff.
- Front page was revised precisely according to the PLOS ONE formatting guidelines.

2. **An overlapping content**

We noticed you have some minor occurrence of overlapping text with the following previous publication[s], which needs to be addressed.

Cheng DR, et al. BMJ Paediatrics Open 2023;7:e001874

https://bmjpaedsopen.bmj.com/content/bmjpo/7/1/e001874.full.pdf.

We have already cited their report in ‘Introduction’. (Note: It was written as UK study but should be Australian study. Sorry for our misunderstanding. It is now corrected in revised manuscript at Line 65.) as shown below:

**[Line 58]** ‘A study in Australia reported that 117 pediatric patients were admitted during the Delta wave (1 June 2021-21 December 2021) compared with 737 during the Omicron wave (22 December 2021-30 September 2022), with the peak number of weekly admissions being 14 for Delta and 53 for Omicron [5]’.

In this report, they documented the upsurge of pediatric patients. Based on their data and others, we analyzed the cause of the upsurge focusing on the viral tropism possibly influenced by the TMPRSS2 receptor. There is overlapping as the same issue but no duplication between two studies.

3. **Budget sources**

In your revision ensure you cite all your sources including your own works, and quote or rephrase any duplicated text outside the methods section. Further consideration is dependent on these concerns being addressed.

Funding is deleted from the front page. It is separately submitted with the revised manuscript.

**Funding:** This work was supported in part by a Grant-in-Aid for Scientific Research on Infection Control and Prevention by the International Platform for Dryland Research and Education, Tottori University. The funders had no role in study design, data collection and analysis, decision to publish, or preparation of the manuscript.

4. **Funding information**

Please note that funding information should not appear in any section or other areas of your manuscript. We will only publish funding information present in the Funding Statement section of the online submission form. Please remove any funding-related text from the manuscript.

Please refer to No.7.

5. **Funder’s role**

Thank you for stating the following financial disclosure: "This work was supported in part by a Grant-in-Aid for Scientific Research on Infection Control and Prevention by the International Platform for Dryland Research and Education, Tottori University." Please state what role the funders took in the study. If the funders had no role, please state: "The funders had no role in study design, data collection and analysis, decision to publish, or preparation of the manuscript." If this statement is not correct you must amend it as needed. Please include this amended Role of Funder statement in your cover letter; we will change the online submission form on your behalf.

Please refer to No.7.

6. **Data set (Minimal data set)**

We note that your Data Availability Statement is currently as follows: All relevant data are within the manuscript and its Supporting Information files. Please confirm at this time whether or not your submission contains all raw data required to replicate the results of your study. Authors must share the “minimal data set” for their submission. PLOS defines the minimal data set to consist of the data required to replicate all study findings reported in the article [<https://journals.plos.org/plosone/s/data-availability#loc-minimal-data-set-definition>]. For example, authors should submit the following data:

- The values behind the means, standard deviations and other measures reported.
- The values used to build graphs.
- The points extracted from images for analysis.

**[Line 297]** The sequences are shown in GenBank accession numbers, LC793397-LC793465 as described in the footnote of Fig 1.

**[Line 286]** The values behind the means, standard deviations for building graphs appearing in Figs 2 and 3 are included as ‘S1 Dataset (XLSX)’ in ‘**Supporting information**’ section after ‘**Conclusion**’ section in the revised manuscript. Excel file of S1 is also attached separately.

7. **Full ethics statement**

Please include your full ethics statement in the ‘Methods’ section of your manuscript file. In your statement, please include the full name of the IRB or ethics committee who approved or waived your study, as well as whether or not you obtained informed written or verbal consent. If consent was waived for your study, please include this information in your statement as well.

It was already included in **‘Methods’ section** as described below. The chairperson has been Dean of Faculty of Medicine, Tottori University. This information was incorporated in the revised manuscript.

**[Line 96]** The present study was approved by the Institutional Review Board represented by the Dean of the Faculty of Medicine, Tottori University, Japan (No.20A138, October 30, 2020) and have conducted from November 1, 2020, onwards.

8. **Table in manuscript**

Please include your tables as part of your main manuscript and remove the individual files. Please note that supplementary tables [should remain/ be uploaded as separate "supporting information" files.

Now the Table 1 appears as a part of the manuscript according to your suggestion.

9. **Consent of participants**

Please provide additional details regarding participant consent. In the ethics statement in the Methods and online submission information, please ensure that you have specified what type you obtained [for instance, written or verbal, and if verbal, how it was documented and witnessed]. If your study included minors, state whether you obtained consent from parents or guardians. If the need for consent was waived by the ethics committee, please include this information.

We have used ‘opt-out’ system to obtain informed consent as described already in‘**Methods’ section**, the part of **Ethical issues including informed consent.** In terms of protection of human rights for minors, we stated that ‘A guardian was selected to protect human rights for a pediatric patient’ as described also in‘**Methods’ section**.

**[Line 103]** Sample collection was carried out after obtaining informed consent from patients through a written website notification and/or poster presentations at the out-patient departments in two hospitals (‘opt-out’ system). It was guaranteed that the freedom to decline participation as a research subject, and refusal to participate would have no negative effect on the person, and that he/she would be able to continue to receive necessary medical cares. Transparency was preserved to the patients for all information and the information was ready to be discarded according to the patient will. Medical records have been accessed only by selected persons on limited occasions. A guardian was selected to protect human rights for a pediatric patient. Only when all the necessary conditions were fulfilled as described above, patient samples were collected and shipped to the laboratory anonymously.

10. **Reference in detail**

Please review your reference list to ensure that it is complete and correct. If you have cited papers that have been retracted, please include the rationale for doing so in the manuscript text or remove these references and replace them with relevant current references. Any changes to the reference list should be mentioned in the rebuttal letter that accompanies your revised manuscript. If you need to cite a retracted article, indicate the article’s retracted status in the References list and also include a citation and full reference for the retraction notice.

We made the ‘Reference’ using PLoS style in EndNote application. I have reviewed again the reference list cautiously. Only one, there was useless gaps in the word of USA. Now the ‘U S A’ was corrected now to ‘USA’ without gaps at **‘Reference’** section, No. 13 as follows:

**[Line 380]** 13. Matsuyama S, Nao N, Shirato K, Kawase M, Saito S, Takayama I, et al. Enhanced isolation of SARS-CoV-2 by TMPRSS2-expressing cells. Proc Natl Acad Sci USA. 2020;117(13):7001-3. Epub 2020/03/14. doi: 10.1073/pnas.2002589117. PubMed PMID: 32165541; PubMed Central PMCID: PMCPMC7132130.

**REVIEWERS' COMMENTS:**

1. **Is the manuscript technically sound, and do the data support the conclusions?**

Reviewer #1: Yes

Reviewer #2: Yes

2. **Has the statistical analysis been performed appropriately and rigorously?**

Reviewer #1: Yes

Reviewer #2: Yes

3. **Have the authors made all data underlying the findings in their manuscript fully available?**

Reviewer #1: No

Reviewer #2: Yes

4. **Is the manuscript presented in an intelligible fashion and written in standard English?**

Reviewer #1: Yes

Reviewer #2: Yes

Thank you for giving approval to the most parts of contents of our research. We agree with the judgement [3. Reviewer #1] for the lacking the Delta variants from pediatric patients. This should be dependent on data given by other studies.

5. **Review Comments to the Author, Reviewer #1:**

Analysis of indicated groups depending specific conditions are valuable for comparing the spreading rate of the Covid-19. The article has some disadvantages such as limited number of samples and population as indicated by the authors. But results of obtained samples support the hypothesis. Additionally:

- In article, Table 1 that has been indicated in lane 194 and 200 was missing.
- Word ‘positive’ has been written mistakenly as ‘postive’ in some part of the article.
- Discussion and conclusion part would be written in more detail and strong.

I agree with the limitation indicated by Reviewer #1, failing in the sampling of Delta variants from pediatric patients because of rare infections caused by Delta variants in the study field. We cannot do the discussion based on Delta variants collected from pediatric patients as described in ‘Discussion’ section.

Table 1 was prepared separately from the main manuscript. It is now incorporated into the revised main manuscript. Thank you for your close attention and pointing out of the typo at line 227. I revised it precisely in the manuscript.

According to your suggestion for more detailed and stronger expressions in ‘**Discussion and conclusion parts**’, an additional sentence is incorporated and deleted unnecessary sentences in the revised manuscript to highlight the main issue of this study as described below:

[**Line 276**] Even so, the above-mentioned epidemiological evidence strongly suggested that the emergence of Omicron variants and those TMPRSS2-independent entry into cells caused the sudden increase of COVID-19 epidemic size among children.

[**Line 282**] The available epidemiological evidence strengthen that the TMPRSS2-dependency of SARS-CoV-2 variants is one of the most crucial determinants influencing the COVID-19 epidemic size among children.

6. **Review Comments to the Author, Reviewer #2:**

The study entitled 'Difference in TMPRSS2 usage by Delta and Omicron variants of SARS-CoV-2: Implication for a sudden increase among children' is descriptive in nature and presents significant findings. With regard of the study "Analysis of epidemic strains from indigenous populations with similar genetic backgrounds in one area showed that the Omicron variants were able to replicate independently in pediatric patients with lower levels of TMPRSS2 on epithelial surface of the respiratory". It is acceptable.

Authors really appreciate that the Reviewer #2 recognized the importance of the study in indigenous populations with similar genetic backgrounds.
